# A New Proposal for Phoneme Acquisition: Computing Speaker-Specific Distribution

**DOI:** 10.3390/brainsci11020177

**Published:** 2021-02-01

**Authors:** Mihye Choi, Mohinish Shukla

**Affiliations:** Department of Psychology, University of Massachusetts Boston, Boston, MA 02125, USA; Mihye.Choi001@umb.edu

**Keywords:** speaker variability, distributional learning, speech perception, phonemic categories, language acquisition

## Abstract

Speech is an acoustically variable signal, and one of the sources of this variation is the presence of multiple speakers. Empirical evidence has suggested that adult listeners possess remarkably sensitive (and systematic) abilities to process speech signals, despite speaker variability. It includes not only a sensitivity to speaker-specific variation, but also an ability to utilize speaker variation with other sources of information for further processing. Recently, many studies also showed that young children seem to possess a similar capacity. This suggests continuity in the processing of speaker-dependent speech variability, and suggests that this ability could also be important for infants learning their native language. In the present paper, we review evidence for speaker variability and speech processing in adults, and speaker variability and speech processing in young children, with an emphasis on how they make use of speaker-specific information in word learning situations. Finally, we will build on these findings to make a novel proposal for the use of speaker-specific information processing in phoneme learning in infancy.

## 1. Introduction

Phonemes differ across languages. English speakers, but not Hindustani speakers, discriminate the *v/w* (vest/west) categories, while Hindustani, but not English speakers, discriminate the daal/ɖaal (d/ɖ; lentils/branch) categories. How does a language learner begin to acquire these linguistic units? It has been proposed that learning phoneme inventories, that is, specific consonants and vowels in a language, occurs during the first year of life [1,2,3]. Early investigations showed that, by 6 months of age, infants are capable of discriminating acoustic tokens corresponding to two different phonemes in a categorical manner. They exhibit such categorical perception of not only their native contrasts, but also non-native speech contrasts. For example, by 6 months of age, American English learning infants can discriminate the Hindustani “daal” from “ɖaal”, a contrast that is not categorically present in their native language input. In this sense, they are *universalists* [3]. Around 12 months of age, however, they become like American English-speaking adults, and fail to make this discrimination, while improving their discrimination of native contrasts; a phenomenon termed “perceptual narrowing” [4,5]. It is important to note that not all phonemes are easily discriminated early in life. There are certain phonemes that cannot be discriminated even if they are present in the ambient language (e.g., see [6] for the fricatives [s] and [z] and see [7] for nasal consonants).

What mechanism explains “perceptual narrowing” in speech perception? Over the last several decades, two major hypotheses have been proposed for phoneme learning in general—minimal pair-based learning and distributional learning (detailed in the next section). Briefly, according to the minimal pair learning account [8,9], infants need to acquire knowledge of minimally contrastive words (e.g., “bear” and “pear”) so that they could attend to the phonetic distinction (e.g., /b/ and /p/). However, the discovery of categorical perception and perceptual narrowing in the first year has led to this theory being disfavored. The other hypothesis, the distributional-based learning account, was proposed by Maye, Werker, and Gerken. Maye and colleagues [10] showed that infants are sensitive to the frequency distribution of phonetic tokens and infer the number of phonemic categories using this information. For example, 6–8-month-olds appear to compute how frequently speech sounds are distributed (e.g., bimodally or unimodally) and then categorize sounds into one or two groups based on the number of modes of the observed distribution over the input tokens.

While these two accounts have driven much other research in this field, there has also been growing evidence that suggests the two accounts may not be sufficient. For the minimal pair learning account, the available data suggest that infants already appear to acquire /b/ and /p/ distinction before they know minimally contrastive words [11]. For the distributional-based learning account, while there is no one clear piece of evidence to contradict this hypothesis, little is known about how frequency distribution is computed in the presence of acoustic variation of tokens. Support for the hypothesis indeed was mostly limited to phoneme learning situations with little acoustic variation, and failed to provide evidence concerning how learners would compute frequency distribution in naturalistic, acoustically varied learning situations.

At the core, speech signals show the *lack of invariance* problem. That is, the speech signal for a given linguistic unit is not invariant, and there is a many-to-one mapping between acoustic signals and a given linguistic unit, like the word “dog”. This problem could result from many different sources. For example, the acoustic properties of a phoneme could be influenced by its location in a syllable or phonetic environment (i.e., linguistic context-dependent variation). The presence of multiple speakers (i.e., speaker variability) is another source that makes the speech signal lack invariance. Adults and children have different vocal tract sizes, and men and women generally differ in their fundamental frequencies (see [12]). Different speech registers (social context-specific variations of a given language, e.g., infant-directed vs. adult-directed speech) or idiosyncratic speaker variation can also lead to differences in how phonemes are produced. For example, [da] and [ɖa] are not contrastive in English and different speakers could consistently produce tokens closer to one or the other.

Listeners, therefore, must be able to find a common phonetic category from the speech signals in the presence of such variation. However, little is known about how a language learner accounts for this variation. Indeed, such variability is widespread in early language acquisition, and phonemic learning situations with little variation are not the typical learning environment. A language learner would encounter multiple people speaking simultaneously, environmental noise, and other distractions (e.g., television) that could increase acoustic variability [13]. Visual cues are also present in the environment, and there can be an infinite number of potential referents [14].

It is possible that the frequency distributions could possibly generate the wrong number of phoneme categories owing to the acoustic variation from multiple speakers. For example, a bimodal distribution may still represent one phoneme in cases where two speakers who have different dialects speak the same phoneme in their own ways (between-speaker bimodal distribution), thus misleading listeners to infer two phonemes. It is also possible that a perceived unimodal distribution may actually be two underlying phonemes if two speakers have a similar pronunciation for different phonemes (e.g., “bet” from one speaker vs. “bat” from another), leading the listeners to infer the wrong number of categories.

It is thus important to understand how listeners deal with speaker variation, and this indeed has been the focus of several recent studies. The overall pattern of results suggests that listeners quickly adapt to speaker-specific variations in the phonetics of the speakers’ productions. Discriminability is not simply weakened in the face of speaker variability [15,16,17]. In adults, tokens that are on a continuum from /d/ to /t/ seem to be more perceived as /d/-like sounds after listeners were exposed to an ambiguous /d/ sound in /d/ containing words [15]. This tendency does not seem to change even when listeners were faced with a new speaker (the effect can be generalized to a new speaker).

Some early work already reported that young infants could discriminate speech sounds despite speaker variability (see [18]), even though the scope of the study is limited in testing a single age group with synthesized auditory stimuli. Furthermore, there have been recent attempts to investigate how young infants deal with such variation in phoneme learning. Bergmann and Cristia in 2018 [19] demonstrated that young listeners have similar variation processing capacities to those shown in adults. A series of papers from Cristia and colleagues have also addressed the issue of phonological acquisition with such variation in infants. Yet, to the extent of our knowledge, there is no study that has investigated how distributional information is utilized when listeners are faced with multiple speakers.

In the present review, we first detail the evidence for the following: (1) adults’ ability to process speech signals in the presence of speaker variability and (2) speaker variability and speech processing in young children. We specifically highlight available evidence for how children make use of speaker-specific information in language learning situations (word learning, for example). We then build on these findings to make a novel proposal that the ability to keep speaker specific-information and to compute speaker-specific distributions in the acquisition of native phonemic categories is within the capacity of young infants. With the issues related to the current phonological acquisition hypotheses [20], we believe that this proposal will contribute to building an integrated framework to explain the mechanisms that underlie this developmental phenomenon in phonemic acquisition.

## 2. Previous Hypotheses: Minimal Pair and Distributional-Based Learning

In the field of classical phonology, phonemes in a particular language are identified using minimal pairs. A minimal pair here refers to a pair of words that are identical aside from one phone in the same position, such as “red” and “led”, but have different meanings. In other words, when a change in the acoustic pattern leads to a change in meaning (e.g., from “red” to “led” or to “ged”), the minimally changed units (the phones [r], [l], and [g]) are considered as phonemes (/r/, /l/, and /g/). The minimal pair hypothesis states that infants must experience that the meanings of two words differ, for example, “bear” and “pear”, and then use this information to attend to the phonetic distinction, “b” and “p” and assign these to separate phonemes (see [21] for an overview). This means that understanding word meanings must precede the phoneme distinction. This is a lexically-driven hypothesis. However, much empirical evidence has pointed out that (1) the earliest age at whichwe can observe a perceptual reorganization in the speech perception domain in infants is as young as 6 months of age for vowel categories [22] and (2) infants might not have large enough vocabularies by the end of the first year of their life, when reorganization is well underway for the consonants of their language. Although we know that they develop their vocabularies between 6 to 8 months of age [23,24,25], these vocabularies are very likely insufficient for a minimal pairs-based phoneme learning account.

**Figure 1:**
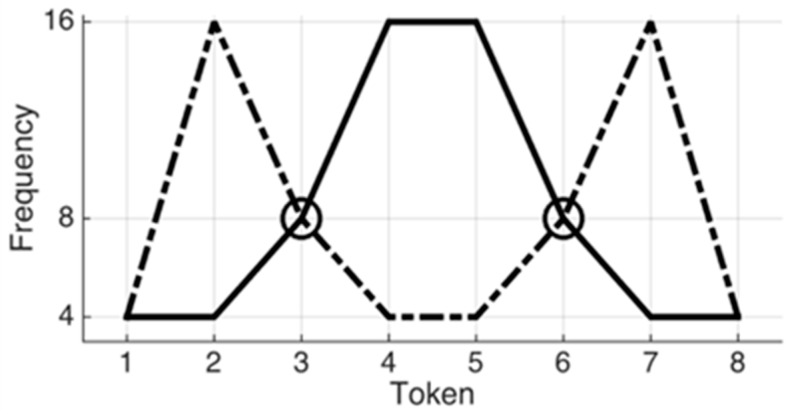
Example of two types of frequency distributions. Dashed line indicates a bimodal distribution, while solid line indicates a unimodal distribution.

Turning to the distributional-based learning hypothesis, infants are proposed to track statistical regularities in their speech input and use this information to build phonemic categories. For example, they compute the frequencies with which the sounds of a specific set of tokens are distributed (e.g., bimodally or unimodally) and infer phoneme categories from the number of modes of the observed distribution [10]. In a series of studies, Maye and colleagues [10,26,27] revealed that infants at 6–8 months of age who were exposed to a sequence of auditory tokens on an eight-step continuum with a bimodal frequency distribution could tell apart the end tokens of the continuum (e.g., Token 1 and 8 in Figure 1). The bimodal distribution had two peaks in the frequency distribution of the tokens (see Figure 1 dashed line, the second and the seventh token are the most frequent sounds among the eight tokens). That is, the bimodal distribution led the young learners to extract two different categories from the continuum. However, infants who were exposed to the same tokens on the same continuum, but with a unimodal distribution (see Figure 1, solid line) failed to show such discrimination to the same test tokens, suggesting that they grouped all the tokens into the same category after the exposure. Support for the distributional hypothesis comes from studies from other domains showing that infants are sensitive to statistics in phonetic discrimination [10], in speech segmentation [28], as well as in word orders in sentences [29].

However, as noted earlier, these studies do not provide an account for infants’ strategies to make use of frequency distributions that vary with the speaker, thus limiting the application of this model to learning situations with very little variation. However, this is not a likely situation that a language learner would encounter. Variations due to different emotional states, variations between children and adults or between men and women due to anatomical/physiological differences, and variations due to different *speech registers* (social context-specific variations of a given language, e.g., infant-directed vs. adult-directed speech) can lead to differences in how phonemes are produced and expose infants to very acoustically varied environments.

Indeed, computing statistical information in the presence of multiple speakers can be a very important task for learners because speaker variability could actually lead listeners to infer the wrong number of phoneme categories. It is possible that the word “bet” produced by one speaker and the word “bat” pronounced by a second speaker may have the same basic acoustic features. It is also possible the word “car” might have substantially different acoustic features when produced by two different speakers. In the frequency distributional account, the former would have one mode in the frequency distribution even though it refers to two different categories (i.e., /e/ and /ae/), while the latter would have two modes (if /a/ of the “car” differs between two speakers) even though it refers to a single category. Such confusion could arise between two speakers of different dialects in the same language. For example, /t/ is realized as an alveolar stop in standard English, but as a dental stop in New York City English. This problem is also increasingly compounded in our ever-growing multicultural society, where the same phoneme might have acoustically distinct productions in speakers of different languages (e.g., an English /p/ versus French /p/). Novice listeners, therefore, must come to learn that alveolar /t/ and dental /t/ are the same phoneme in this case, even though the acoustic features might indicate the different number of phoneme categories.

Fortunately, recent evidence points to sophistication in infants’ learning strategies. In terms of infants’ sensitivity to speaker variability, there is now evidence showing that they are capable of storing context-specific information and applying this information towards word learning. For example, when presented with a unimodal distribution between English [ba] and [da] (indicating a single underlying phoneme), 6-month-old English-learning infants nevertheless appear to induce two categories when the tokens along the continuum are accompanied with faces displaying two different mouth shapes corresponding to productions of a [ba] and a [da], but not when the faces only display a [ba] or a [da] mouth shape [30]. Additionally, 9-month-old English-learning infants only induce two underlying categories when the two phonetic categories of Hindustani [da] and [ɖa] are consistently paired with two different novel objects, but not when the two categories are randomly paired with both of the two novel objects [31]. The two studies mentioned above showed that phonetic category learning is constrained by contextual information.

Armed with this evidence for infants’ sensitivity to speaker variability, in the next section, we propose that infants are capable of computing speaker-specific distributions and further hypothesize that multiple relevant and contextual cues, which could make the learning situation more complicated, might instead make it easier for infants to find phonemes.

## 3. A New Proposal: How Do Learners Deal with Speaker Variability in Phoneme Learning?

The first year of life is assumed to be a critical period for phonological learning [3,18,32]. At around 9 months of age, infants begin to attend to their native phonology, while they become less sensitive to speech sounds that are not utilized as linguistic units (phonemes) in their native language (a loss of previous competence to non-native phonemes, a.k.a., perceptual narrowing) [33,34].

In the distributional-based learning account, the presence of a unimodal distribution (modal tokens in linguistic input fall somewhere between [da] and [ta], for example) leads to an interpretation of a single phoneme category within the continuum, whereas a bimodal distribution is interpreted as evidence for two phoneme categories. As stated earlier in this paper, such a strategy may be misleading. A bimodal distribution in linguistic input may indeed indicate different phonemic categories within the language, but may also indicate speaker differences in production (i.e., accents). In order to systematically learn the appropriate phoneme contrasts despite such variation, infants might rely on a multitude of cues in addition to the distribution of acoustic tokens.

One way to overcome this confusion with the distributional information in inferring phonemic categories may be to compute speaker-specific distributions. For example, there is a listener who computes whether a variety of /r/ pronunciation from one speaker is distributed unimodally or bimodally for that speaker and builds *speaker-specific distributional* representations. It can prevent the listener from inferring the wrong number of phonemic categories owing to speaker variability. The listener can make an inference that only those distinctions that are bimodal within individual speakers indicate a phonemic distinction. There are important findings reporting that infants can extract speaker-specific features from short exposure and apply this knowledge to language learning. A recent study determined that 10- to 12-month-olds can build knowledge of speaker-specific variants and use the knowledge to make an inference of mapping appropriate objects [35]. There is much research that has also demonstrated that it is within infants’ capacity to learn speaker-specific representations, leading to our hypothesis that it may be also possible for infants to compute speaker-specific distributions.

Our hypothesis that infants are able to compute speaker-specific distributions is centered around this assumption that infants rely on a multitude of cues in phonemic learning because they use not just distributions of tokens, but also their relationship to acoustic evidence that distinguishes two speakers. When we say that infants compute speaker-specific distributional representations, we are implicitly assuming that there are at least two separate computations: distributional computations and computations that link distributions to individual speakers. While speaker identity could be inferred from the acoustic characteristics of speech tokens, there is no reason to suspect that speech is the only source for indexing distinct individuals; for example, infants could use visual information in identifying distinct individuals.

In fact, prior research suggests that statistical learning is constrained by other cues, including visual ones. Teinonen and colleagues [30] showed that English learning infants at 6 months of age were capable of using visually presented faces to learn two different categories in a unimodal frequency distribution. Moreover, Yeung and Werker found 9-month-old English learning infants could hear a difference between Hindi phonetic contrast (e.g., a dental alveolar stop [da] and a retroflex alveolar stop [ɖa]) only when the sounds were consistently paired with two different visual cues [31]. Recent work also suggests contributions from computing audio–visual correspondences (see [36,37]), and perhaps even sensory–motor correspondences [38].

With the evidence presented in the next section that listeners, in both adults and infants, are capable of dealing with speaker variability and this ability is systematic, we infer that (1) young listeners may be able to compute speaker-specific distributions as they could build speaker-specific representations and (2) computing speaker-specific distributions would also interact with other contextual cues. Taken together, these support our novel proposal that infants are possibly capable of using multiple cues to infer phonemic categories when they learn their native phonology.

### 3.1. Evidence from Adults

Traditional views regarding how listeners solve the lack of invariance problem have focused on perceptual normalization processes [39,40]. The general assumption in these accounts is that, in the mental dictionary, each word is stored with only one canonical phonetic form, and speech signals that do not match the canonical form would be filtered. That is, idiosyncratic features of different speakers would not be regarded as necessary for further processing and be discarded. For example, Mullennix, Pisoni, and Martin showed perceptual performance in recognizing words decreased when the voice of the speaker changed across trials [41]. In their study, participants showed higher performance on identifying monosyllabic words after they heard the words—CVC format (i.e., consonant—vowel—consonant syllables) from a single talker than multiple talkers. In a subsequent study, they found that listeners had difficulty ignoring speaker variability in word recognition [42]. To summarize these and other results, this early literature seems to show that speaker variability decreases listeners’ ability to process speech signals [43,44,45].

However, this point of view that speaker variability is thought of as an uninformative cue in speech processing began to be challenged by the evidence that speaker-specific features are not discarded. For example, in Lively and colleagues’ work, Japanese listeners were trained on words containing either English /r/ or /l/ and were asked to identify whether the word contains /r/ or /l/ [16]. Importantly, the words were produced by multiple speakers. The results showed that not only did Japanese listeners have increased performance to discriminate English /r/ and /l/ after training, but also they could recognize English /r/ or /l/ embedded in new words pronounced by a new speaker. This was not observed in the other group of participants who were trained with a single speaker.

Additionally, recent literature indicates that listeners are not only sensitive to speaker variability, they are also capable of storing speaker-specific variation [46,47]. Kraljic and Samuel [15] showed that listeners’ perception of a target token was influenced by its lexical context. For example, listeners tended to perceive tokens on a continuum from /d/ to /t/ as more /d/-like after being exposed to an ambiguous d-t sound (mixture of /d/ and /t/) in /d/ containing words (further, exposure to an ambiguous d-t sound in /t/ containing words led listeners to perceive more /t/ tokens on the continuum). This learning effect was still observed for a different voice. Their following study [46] further determined that this perceptual learning occurs in the speaker dimension as well. Listeners had lexical training with two speakers and the two speakers had systematically different pronunciations of fricative consonants. One speaker produced a sound in between /s/ and /ʃ/ in /s/ containing words and the other speaker produced the same sound, but in /ʃ/ containing words. Listeners tended to perceive the continuum differently according to each speakers’ feature; that is, they categorized the continuum as more /s/-like when it was produced by the speaker who spoke the midway sound in /s/ containing words, while they perceived the same tokens on the continuum as /ʃ/ when they were produced by the other speaker who had the tokens in /ʃ/-containing words. Listeners were able to maintain representations of multiple speakers and so their perception of the tokens on the continuum was adjusted according to their experience of each speaker. Interestingly, listeners did not show such a pattern of perception in stop consonants. This shows that the experience of multiple speakers can lead listeners to build speaker-specific representation and use speaker-specific information in learning phonemic categories; although, at least in adults, this process might interact with the class of phonemes.

Exposure to multiple speakers or experience with new speakers can sometimes also help listeners to learn better. For example, Nygaard and Pisoni [48] found that exposure to words spoken by multiple speakers improved listeners’ intelligibility to the words in novel sentences. Furthermore, listeners who had experience with multiple speakers from different regions could recognize each region according to the speaker’s dialects, but this learning did not occur when the listeners had a single speaker exposure of each region, suggesting that the advantage of experience multiple speakers on perceptual learning appears to be observed with dialects as well [49].

Overall, the findings above suggest that listeners can develop speaker-specific representations as well as linguistic context-dependent representations in phonemic processing [16,46]. This ability to deal with speaker variability seems very sophisticated and robust. Adult listeners do not simply discard the variation. Rather, they keep speaker-specific representations and utilize them to adapt to new learning situations. To build speaker-specific representations (systematic patterns of each speaker), surprisingly, they do not need considerable amounts of exposure.

### 3.2. Evidence from Toddlers

A similar pattern of results shown in adults has been observed with younger populations. There is clear evidence that young populations do not just discard speaker-specific variation (such as the speaker’s voice characteristics). It was already evident from studies in the 1980s that infants could remember fine acoustic details in distinguishing speakers. For example, DeCasper and Fifer [50] showed that newborns could remember a specific voice (e.g., mother’s voice); newborns seemed to prefer their mother’s voice to another female’s voice. Young learners could also distinguish their mother’s voice from other voices [51]. These findings suggest that they can in principle build speaker-specific representations.

In word recognition studies, toddlers appear to adapt speaker-specific features as quickly as adults do and they could recognize familiar words in new accents [52,53]. Van Heugten and colleagues [53] investigated whether Canadian English-learning toddlers recognize familiar words spoken by an unfamiliar Australian accent. After the toddlers were exposed to a child-friendly story, The Very Hungry Caterpillar, produced by a speaker with an unfamiliar accent (an Australian English-Cantonese bilingual female speaker), the toddlers were tested on their comprehension of words spoken either by the same or the other non-native speaker. During the test, two pictures (a target and a distractor) were presented on the screen side by side and a familiar word, such as “cake” was played. Twenty-five-month-old toddlers recognized the words correctly for both familiarized and non-familiarized non-native speakers (a male native speaker of Cantonese).

For toddlers at 2 years of age, it seems even possible to learn new words from multiple speakers. Schmale, Cristia, and Seidl [54] demonstrated that 24-month-old toddlers could always learn new words from either a single speaker or multiple speakers with a short exposure to the speaker(s). This ability seems to be maintained even when the accents were non-native (e.g., Spanish-accented). Potter and Saffran reported similar observations in word recognition in 18-month-olds [52]. Surprisingly, the toddlers had better performance at recognizing words for an unfamiliar accent after they were exposed to brief passages of multiple speakers with multiple accents (a mix of Australian, Southern-American, and Indian accents) than when they were exposed to multiple speakers of a single accent.

These results demonstrate that, by the second year of life, toddlers seem not to have difficulty in learning new words as well as recognizing familiar words even if the words are produced by unfamiliar accents. It is also evident that experience of more variability may indeed be advantageous for recognizing words in unfamiliar accents. Although there are mixed results reported in terms of the age that toddlers can take advantage of speaker variability in word recognition, it appears that both native/non-native accents and speaker variability contribute to the process. For example, while van Heugten and colleagues found that 20-month-olds failed to recognize the familiar words in the presence of a speaker with an unfamiliar accent [53], Potter and Saffran reported that younger infants (18 months of age) could recognize words in unfamiliar accents after having multiple accent exposures [52]. Schmale and Seidl [55] also indicated that 13-month-olds appeared to have no trouble recognizing familiar words embedded in sentences when the voice characteristics of the speaker changed (i.e., one native speaker during familiarization and another during the test) or accent changed (i.e., change from a native speaker to a non-native speaker). In contrast, younger, 9-month-old infants in this study could not succeed in this task when the speakers changed from native to non-native accent, and only succeeded for speaker changes in the native accent.

Young children are also able to apply speaker-specific knowledge to learning situations [35,55,56,57]. White and Aslin found that infants as young as 19 months of age could quickly adjust to a speaker’s unique pronunciation—the speaker pronounced a vowel [a]-containing familiar words, such as dog, as a vowel [æ]- containing words (i.e., a shift from [a] to [æ])—and correctly map the vowel of the switched words to their proper referents based on their experience with the speaker [57]. Weatherhead and White further suggested that 10–12-month-old infants could not only remember speaker-specific features, but also utilize this information in learning new labels [35]. In the study, the infants heard two speakers whose pronunciations of a certain vowel (e.g., front vowels) were systematically different during exposure. For example, one speaker had higher front vowels compared with the other speaker (“t/I/pu” vs. “t/E/pu”) and infants were exposed to the two speakers saying the two words with an object. If infants could learn these productions are systematically different across two speakers and learn that the different pronunciations are speaker-specific variants, they could infer that the word “t/E/pu” from the speaker who said “t/I/pu” refers to the new object, while the same word (“t/E/pu”) from the other speaker (who said“t/E/pu” during the exposure) indicated the same object from the training. The results confirmed the prediction; infants showed longer looking times to the untrained object (i.e., new object) after they heard that the “t/E/pu” speaker said “t/I/pu”, but showed the reversed looking pattern (i.e., looked longer to the trained object) when the “t/I/pu” speaker said “t/I/pu”.

While there is no direct observation reported, these findings support our hypothesis that young infants could keep speaker-specific variants and this ability can be applied to computing speaker-specific distribution.

It is important to note that this proposal can explain the phoneme acquisition in the presence of multiple speakers not only in monolinguals, but also in bilinguals. In fact, language learners who are raised in multicultural societies are more likely to be exposed to increased variability because of the possible presence of non-native accented speakers (see [16,58]). The classic distributional hypothesis may be difficult to apply in this case where the same phoneme might have acoustically distinct productions in speakers of different languages (e.g., an English /p/ versus French /p/).

In bilingual native phonetic discrimination, there is one distinct pattern that is not observed in monolingual development, that is, the U-shaped developmental pattern. For instance, while bilingual Spanish–Catalan infants were able to discriminate a Catalan vowel contrast /e/-/ɛ/ at both 4 months and 12 months, they failed to do so at 8 months (U-shaped pattern). For Catalan and Spanish monolinguals, while Catalan monolingual infants maintained the sensitivity to the contrast at 12 months because it is their native, Spanish monolingual infants no longer discriminated the contrast at the same age [59]. There is also similar evidence found in French and English bilingual infants [60].

We suggest that this short-term delay in bilinguals may be related to their extensive use of other cues, such as audiovisual speech cues. Pons, Bosch, and Lewkowicz found that both 8-month-old Catalan and Spanish monolinguals exhibited longer looking times at the speaker’s mouth than the eyes in response to both native and non-native speech, but 12-month-old monolinguals looked longer at the mouth only to nonnative speech. However, bilinguals always looked longer at the speaker’s mouth for both native and nonnative speech at all ages [61]. If it is true that infants take more advantage of other resources (e.g., visual information from speakers) when their input language has high variability, then our proposal that infants can compute speaker-specific distribution in learning phonemes also seems to be plausible with bilinguals.

## 4. Conclusions: An Enriched View of Phoneme Acquisition

A rich psycholinguistic literature with adults has revealed that adults can rapidly adapt to a variety of speaker-specific variations [26,62,63]. We presented evidence that infants are also sensitive to acoustic differences that mark both language-relevant differences (e.g., different vowels) and accentual differences (see [64]). In addition, there is evidence that infants can integrate visually presented, socially relevant information about speakers with phonetic distributional information to make inferences about phonemes. For example, ter Schure, Junge, and Boersma exposed 8-month-old Dutch infants to English vowel contrasts presented by talking faces and found longest looking to the mouth only in the bimodal condition [37]. Moreover, phonetic, distributional information interacts with visual information about potential referents. Yeung and Werker found that 9-month-old English-learning infants, who typically cannot discriminate auditory tokens for a continuum between [da] and [ɖa], were able to do so when, in a familiarization phase, tokens from the two halves of the continuum were consistently paired with one of two distinct, novel objects [31]. The ability to utilize visually presented, socially relevant information about speakers indicates that infants can extract indexical information about speakers, potentially leading them to compute speaker-specific distributions.

In this project, we suggest that various aspects of language learning can be seen as inferential processes that weigh several sources of information. This proposal also infers that learning phonemic inventories would benefit from the social context in which it occurs and it could explain the rapid narrowing of phonemic categories in the face of acoustic variation that sometimes signals underlying phoneme categories and sometimes not.

What might be the driving force behind using social (auditory or visual) information to guide phoneme learning? Here, we borrow ideas from Relevance Theory and Natural Pedagogy (see [65]). We do so by first noting that, even though the majority of previous studies investigating early language acquisition have done so in experimental contexts that are devoid of any social cues, a study by Kuhl, Tsao, and Liu [66] found that, in the absence of contingent social cues (presentation of auditory material via a live person versus a recording of that person), distributional learning appeared to completely disappear. This observation dovetails with Natural Pedagogy, which aims to understand how infants acquire generic information from specific observations. It proposes that humans are specifically adapted for the transmission of generic information—teachers provide specific ostensive cues that accompany instances where they would like the learner to infer generic information, and infants are sensitive to such cues, and use their presence to make inferences that lead to the acquisition of generic information. For example, if an infant sees Bob looking disgustedly at a glass of a green liquid, the infant could either infer that Bob does not like that green liquid (Bob-specific interpretation) or that green liquids are bad for us (generic interpretation). In a series of studies, it has been shown that infants make generic inferences when these are accompanied by ostensive-communicative cues such as direct eye gaze, contingent interaction, ostensive pointing, or human speech [67].

Phoneme inventories and words are also examples of generic knowledge—if Arya and Sansa speak the same language, we can expect that Arya’s phonemic description of the word “bottle”, and what she intends by that word, generalizes to Sansa. Considering Natural Pedagogy as an appropriate framework suggests that ostensive cues might modulate how infants make inferences when acquiring phoneme categories and words. In fact, there is a slew of evidence that demonstrates that infants, even in their first year of life, consider the perspective of other agents (e.g., [68]), and use ostensive cues as a guide to determine categorization and generalization (see [69,70]). In a recent study, Kovács, Téglás, Gergely, and Csibra found that 12-month-olds were more likely to assign ambiguous objects (e.g., a plate that can turn into a cup) to a demonstrated category, when the demonstrator looked directly at the infant; direct gaze is another ostensive cue [71]. We believe that infants are capable of considering the linguistic role played by phonemes and words as meaningful categories that serve the social-communicative goals of language.

Perhaps, the implications of this novel framework are not limited to phoneme learning, but are, in principle, widely applicable to all of language. This idea, therefore, can hold promise to transform how we view learning categories in all aspects of language, whether these are in phonology, syntax, or semantics, as outcomes of social-communicative inferences.
